# Understanding X-ray Photoelectron Spectra of Ionic Liquids: Experiments and Simulations of 1-Butyl-3-methylimidazolium Thiocyanate

**DOI:** 10.1021/acs.jpcb.2c06372

**Published:** 2022-12-01

**Authors:** Ekaterina Gousseva, Scott D. Midgley, Jake M. Seymour, Robert Seidel, Ricardo Grau-Crespo, Kevin R. J. Lovelock

**Affiliations:** † Department of Chemistry, University of Reading, ReadingRG6 6DX, U.K.; ‡ Helmholtz-Zentrum Berlin für Materialien und Energie (HZB), Berlin14109, Germany

## Abstract

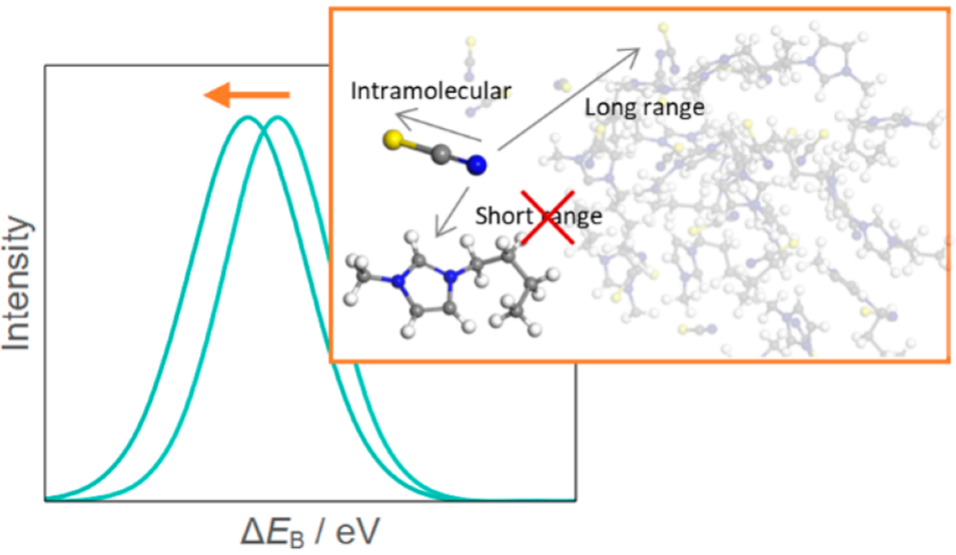

We demonstrate a combined experimental and computational approach to probe the electronic structure and atomic environment of an ionic liquid, based on core level binding energies. The 1-butyl-3-methylimidazolium thiocyanate [C_4_C_1_Im][SCN] ionic liquid was studied using ab initio molecular dynamics, and results were compared against previously published and new experimental X-ray photoelectron spectroscopy (XPS) data. The long-held assumption that initial-state effects in XPS dominate the measured binding energies is proven correct, which validates the established premise that the ground state electronic structure of the ionic liquid can be inferred directly from XPS measurements. A regression model based upon site electrostatic potentials and intramolecular bond lengths is shown to account accurately for variations in core-level binding energies within the ionic liquid, demonstrating the important effect of long-range interactions on the core levels and throwing into question the validity of traditional single ion pair ionic liquid calculations for interpreting XPS data.

## 1 Introduction

Ionic liquids (ILs) are liquids composed exclusively of ions. Their interesting potential properties, including large electrochemical windows, wide liquid ranges, tunability, and low melting points,^1,2^ make then desirable for a range of applications, from catalysis to batteries.^3−7^ Macro- and mesoscopic properties, unique to each IL, are determined by interactions between the cation and anions.^8^ A thorough study of molecular-level interactions in ILs could lead to a method to predict structure, properties, and reactivity^9,10^ and eventually suitability for specific applications. The donation of electron density from anion to cation, often termed charge transfer, has been debated in the IL literature, along with the importance of ion polarizability.^11,12^ The distance dependence of electronic cation–anion interion interactions for ILs, particularly important for understanding the dynamics of ILs, is currently unclear. Furthermore, the range of electronic environments present in the IL has been probed computationally, but not compared to experimental data.^13,14^

X-ray photoelectron spectroscopy (XPS) is a very useful tool to understand these interactions. XPS has traditionally been used on solid or gaseous samples, due to the required ultrahigh-vacuum conditions (UHV).^15,16^ XPS can, however, be applied to ILs as they exhibit very low vapor pressure and therefore are part of a limited group of liquids that can be studied using standard UHV XPS apparatus.^17−19^ XPS has been used for ILs to probe both surface geometric structure (e.g., by varying the IL surface detector angle)^20−22^ and bulk electronic structure.^1^ The study of ILs via XPS offers many opportunities, but also faces several obstacles.

Core-level binding energies, *E*_B_, can be used to understand the electronic structure of ILs as *E*_B_ is the difference between the ground state and an excited state with a core hole.^23,24^*E*_B_ shifts are caused by valence electron behavior, which in turn is affected by the chemical environment around the ion. The position of the core level in the ground state, relative to the vacuum (or at times the Fermi level for experimental data), determines what is called the initial-state (IS) effect in *E*_B_. The ground-state electronic structure is related to the atomic environment, i.e., bonding and interactions. When an electron is photoemitted from the core level, the other core and valence electrons relax. The magnitude of this relaxation affects the *E*_B_ value and is called the final-state (FS) effect.

Interion electronic effects of the anion on the cation have been demonstrated using XPS; however, whether this effect is due principally to IS or FS effects is unclear.^25^ It is usually assumed for XPS of ILs that IS effects dominate the measured *E*_B_.^25−65^ It is an important assumption because, if correct, it means that experimental *E*_B_ shifts give valuable clues about the ground-state electronic structure of ILs. Many studies have been performed under this assumption, relating *E*_B_ of core levels to atomic charge, oxidation state, or electronegativity, to understand and potentially make predictions on structure and reactivity.^25−65^ However, this assumption has so far proved impossible to establish exclusively using experimental methods; core-level *E*_B_ values have been compared to shifts in near-edge X-ray absorption fine structure (NEXAFS) spectroscopy edge energies and Auger parameter values to try to determine the relative influence of IS and FS effects in XPS for ILs.^66,67^

The combination of experimental *E*_B_ and theoretical models to study ILs has had limited use to date. It has been applied to study partial charges and models of a single ion or a pair (one anion with one cation).^25,64,65,67,68^ Two studies reported calculations on larger model systems (ion “clusters”, up to eight ion pairs), but comparisons were only made for valence levels, not for core levels.^69,70^ Comparisons of core *E*_B_ to calculated atomic charges have also been made, which are based on the assumption that IS effects dominate *E*_B_ shifts.^25,64,66,67,69^*E*_B_ of core levels have been calculated for ILs,^65^ but rarely are core holes explicitly included and only on small scale systems such as lone ions or ion pairs.^38,68^

The XPS signal from an IL arises from contributions from a distribution of *E*_B_ values that reflects the range of chemical environments coexisting in the IL. Thus, the broadening of an experimental XPS core level peak has the potential to give information about the geometric structure of a sample.^71^ The full width at half-maximum (FWHM) of a measured core-level peak will contain contributions from a range of factors, including X-ray source and analyzer resolution from the apparatus,^72^ charging,^73^ core-hole lifetime,^74^ and sample geometric structure effects.^75^ For XPS of liquids, only water FWHMs have been widely published; the structural disorder contribution to the FWHM for liquid phase water and ions solvated in water is around 1.0 eV.^75,76^ The experimental FWHM was interpreted in relation to the liquid phase geometric structure, e.g., the hydrogen bonding in liquid water.^76^ For ILs, no investigations of the structural disorder contribution to the FWHM have been made. Developments in *ab initio* molecular dynamics (AIMD) have seen improvements in speed and accuracy,^77^ which has allowed its use in the simulation of ILs.^77−92^ Despite this, no theoretical studies of core levels in bulk liquid phase with explicit ions have been performed on ILs, to the best of our knowledge. A combined approach is required, including computer simulations validated by experimental data, such as peak positions and broadening.

**Figure 1:**
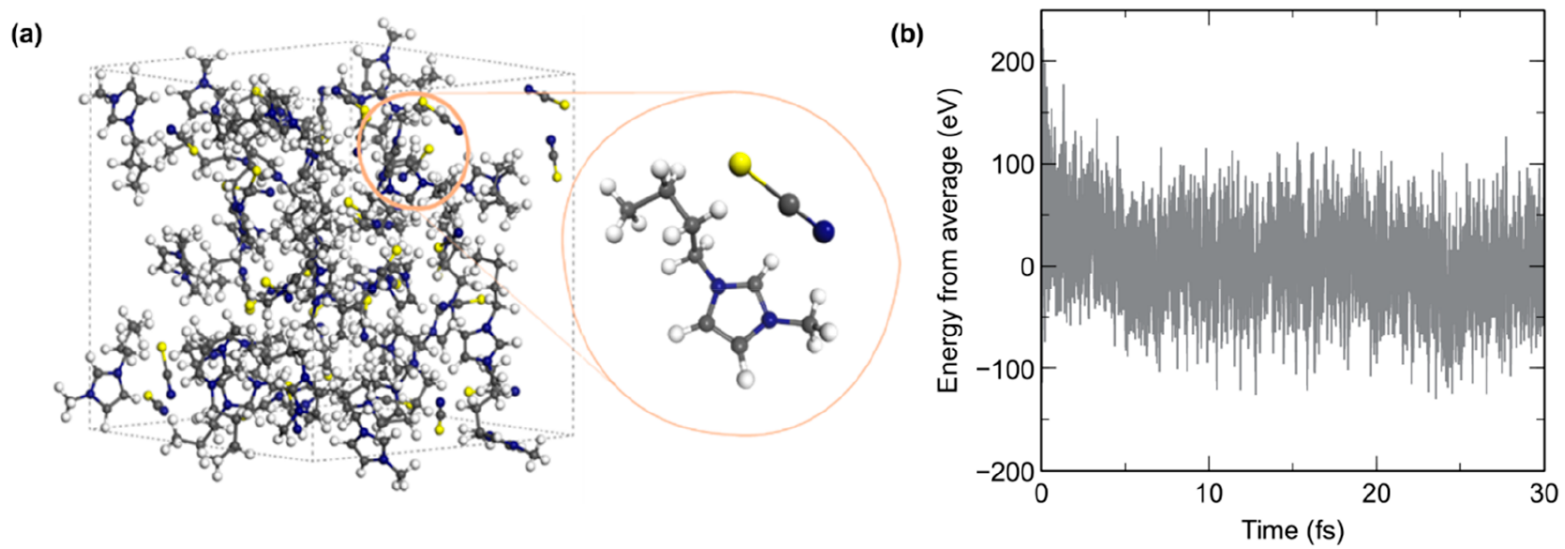
(a) Simulation cell used in this work, which is repeated under periodic boundary conditions. The cell consists of 32 pairs of [C_4_C_1_Im][SCN] to create a disordered liquid phase. (b) Potential energy of the system at each time step over the course of the AIMD calculation. A configuration for further core-hole calculations was taken at a step with average energy to produce 32 conformers.

In this work, we present a theoretical study of core-level *E*_B_ values and distribution in a bulk IL system, [C_4_C_1_Im][SCN]. Experimental XPS data are available for [C_4_C_1_Im][SCN] in the literature.^66,67^ AIMD is used here to simulate a bulk model of this IL, which was chosen due to desirable properties of both the anion and the cation. [C_4_C_1_Im]^+^ is one of the most commonly studied cations, and further understanding of its behaviors will be an asset in several areas of research. Its relatively small size is convenient for expensive AIMD calculations. The anion, [SCN]^−^, is also small. The negatively charged S and N atoms potentially allow for hydrogen bonds to be created in the bulk system.^66,67^ [SCN]^−^ is particularly interesting as it is a pseudohalide, yet [C_4_C_1_Im][SCN] has one of the lowest viscosities among ILs,^93^ in contrast to [C_4_C_1_Im][PF_6_], for example.^94^ Core *E*_B_ values were calculated using density functional theory according to two approximations: (i) including IS effects only (we call this the IS approximation) and (ii) including both IS and FS effects via explicit consideration of the core hole (we call this the FS approximation). We demonstrate an excellent match between experiment and calculations. We show that IS effects are the major contributor to experimental core level *E*_B_, a significant contribution to the sphere of researching ILs using XPS methods. Variation of *E*_B_(core) was assessed in relation to a range of interactions, which is only possible in a calculation of the bulk liquid, as opposed to considering only single ions or ion pairs. A model that describes the variations of *E*_B_ within the IL is presented. The structural disorder contribution to FWHM was compared between theory and experiment.

## 2 Methods

### 2.1 X-ray Photoelectron Spectroscopy (XPS)

Liquid jet XPS measurements for K[SCN] in water were performed at the U49/2 PGM-1 beamline (SOL^3^PES end-station) at the BESSY II electron storage ring.^95^ The SOL^3^PES experimental setup is equipped with a Scienta Omicron R4000 HIPP-2 hemispherical electron analyzer. K[SCN] (Sigma-Aldrich, purity ≥99.0%) was dissolved in ultrapure water. The mole fraction of K[SCN] in water was *x* = 0.01, i.e., 0.5 M. Nonresonant XPS regions were recorded at *hν* = 700.0 eV. The pass energy was 100 eV. The angle between the polarization axis of the incoming photon beam and the electron analyzer was 54.7° (magic angle geometry). All nonresonant XP spectra were fitted using the CASAXPS software. Fitting was performed using a Shirley background and GL30 line shapes (70% Gaussian, 30% Lorentzian). Photoelectron spectra *E*_B_ for [C_4_C_1_Im][SCN] were effectively charge referenced to the literature value of *E*_B_(C_alkyl_ 1s) = 289.58 eV (which corresponds to alignment with vacuum).^96^ Photoelectron spectra *E*_B_ for K[SCN] in water were charge referenced to *E*_B_(N_anion_ 1s) = 402.37 eV, as *E*_B_(N_anion_ 1s) = 402.37 eV for [C_4_C_1_Im][SCN] when charge referenced to *E*_B_(C_alkyl_ 1s) = 289.58 eV. Experimental data for all [C_4_C_1_Im][SCN] measurements were taken from an earlier publication by our group,^109^ where all the experimental details may be found.

### 2.2 *Ab Initio* Molecular Dynamics (AIMD)

A 32 ion pair model of [C_4_C_1_Im][SCN] with a density of 1.07 g cm^–3 ^^97^ was simulated using AIMD with the Quickstep code in CP2K, based on the Gaussian and plane waves method (GPW) and using the direct inversion in iterative subspace (DIIS) technique. After pre-equilibration using the classical force field DREIDING, the AIMD simulation was run for 30 ps with a time step of 1 fs. The potential energy variations were equilibrated after <10 ps of AIMD. This simulation was performed at 398 K controlled by a Nosé thermostat in the NVT ensemble. The PBE functional^98^ was employed, with D2 corrections by Grimme^99,100^ to account for dispersion interactions. A configuration at the end of the simulation, with energy close to the average, was extracted to carry out core-level calculations. An increased temperature of 398 K reduces viscosity and allows for equilibrium to be achieved faster, thus reducing the computational cost of the calculation while remaining in a range safe from thermal decomposition.

### 2.3 Core-Level Calculations in Bulk Ionic Liquid

Calculations of *E*_B_(core) were performed in the Vienna Ab Initio Simulation Package (VASP).^101^ A snapshot configuration with an energy close to the AIMD average was chosen to calculate the distribution of *E*_B_ values across the 32 ion pairs. The core-level calculations also used the PBE exchange correlation functional employed for the AIMD. The core–valence electron interactions were described using projector augmented wave (PAW) potentials.^102,103^ The number of plane waves in the basis set expansion of the wave functions was chosen by setting the kinetic energy cutoff to 400 eV. All core level energies of the system were calculated in this same configuration, for IS and FS. Test calculations showed that the distribution of binding energies was not significantly affected by choosing or adding other configurations.

The IS approximation to calculating *E*_B_(core), as defined in this work, is obtained from the Kohn–Sham (KS) orbital energies. The KS orbital energies are calculated after a self-consistent calculation of the valence charge density. No core hole is produced, and therefore any core-hole-related effects are omitted. Only IS effects influence *E*_B_ and the orbital energies, or core levels (*CL*), are converted to *E*_B_ simply, by
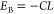
1These values, as obtained from VASP, are aligned with an internal energy reference, so only relative values are meaningful. Here, we shifted the calculated core-level values to match experiment, as explained below.

In contrast, for the FS approximation, the calculation involves creating a core hole explicitly. In the FS method used in VASP, it is assumed the nuclei are static, due to the time scale of the excitation of an electron. Additionally, the other core electrons in the atom are not allowed to relax once the core hole has been created. This may create a slight error, specifically in relation to a lack of lifetime broadening of the resultant peak. The energy extracted is the total energy of the system, including the core hole. This means that the calculated energy is again useless as an absolute energy, and only relative energies can be used. In this work, we have aligned the average FS energy with the average IS energy, to give FS values that are comparable with experiment. This method provides an internal comparison of values; i.e., the absolute value has no inherent meanings, other than its relation to other *E*_B_ values. Absolute energies were not considered, but rather the difference between energies (Δ*E*_B_).

### 2.4 Molecular Calculations

Test calculations of isolated [SCN]^−^ anions were performed using Gaussian16.^104^ These single point energy calculations were performed with the 6-31g(d, p) basis set^105,106^ and the B3LYP functional.^107^ These tests were designed to separate intraion from interion contributions to *E*_B_. Intraion interactions were characterized by the lengths of S–C and N–C anion bonds. One bond was kept constant while the other was modified. The constant bond length was determined by taking an average of the 32 bond lengths in the configuration. For S–C this was 1.65 Å, and for N–C it was 1.20 Å. The same method in Gaussian16, as above, was used to optimize the ions with the second bond length varied at an interval of 0.02 Å, and the *E*_B_ was extracted from orbital energies. We studied short-range interactions by extracting radial distribution functions (RDFs) and visually assessing each anionic environment.

### 2.5 Gaussian–Lorentzian Peaks

A Gaussian–Lorentzian Product (GLP) function is one of several types of functions used to fit peaks in experimental XPS measurements.^108^ XPS peaks are typically expected to be Lorentzian, but due to the various sources of broadening, this shape is distorted and compensated for by including Gaussian mixing in the function. To form a peak from the 32 data points for each core level, eq 2 was applied to the values, where the mixing parameter, *m*, was set to 0.3 as in experimental peak fitting. Values 0 and 1 are pure Gaussian and pure Lorentzian, respectively. The function width, *F*, was set at either 0.7 or 1 eV for high resolution and survey scan peaks, respectively.
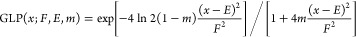
2In the investigation of ILs using XPS, survey scans are typically used to determine the presence of impurities of the sample. They are measured to see what elements are present, and their abundance, using a high pass energy and low resolution. These settings result in greater apparatus broadening contributions to the peaks. High-resolution scans are measured with low pass energy. These are used to identify chemical states and typically have lower broadening than survey scans. Calculated peaks were slightly shifted in position and normalized by intensity for improved comparison with experiment. Values were shifted by +21.2 and +27.0 eV and intensities corrected by the height of the C 1s peak.

## 3 Results and Discussion

### 3.1 Initial-State vs Final-State Approximations to the Core-Level Binding Energies

**Figure 2:**
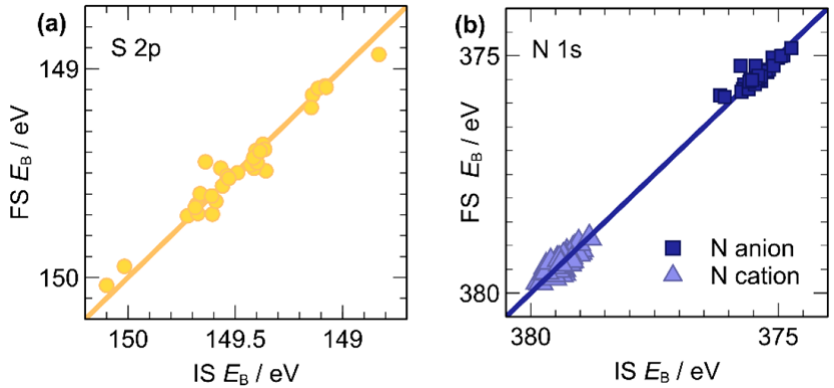
Linear correlations between binding energies calculated in IS and FS approximations for (a) *E*_B_ (S 2p) and (b) *E*_B_(N 1s). The *y* = *x* line is plotted as a guide. *R*^2^ = 0.95 (a), 0.77 (b, anion), and 0.74 (b, cation).

A comparison between calculated binding energies in the IS and FS approximations was performed to identify the dominant effects. Because the calculation in the FS approximation includes both IS and FS effects, a strong positive linear correlation (Figure 2) is reasonable confirmation that FS effects are minor, and therefore IS effects are the primary influence on *E*_B_(core). *E*_B_(S 2p) values calculated in the FS approximation showed strong correlations with the corresponding values calculated in the IS approximation (*R*^2^ = 0.950). Correlation between results in the IS and FS approximations was also good for *E*_B_(N 1s) (*R*^2^ = 0.995), although the correlation is weaker if N_anion_ and N_cation_ are considered separately (*R*^2^ = 0.77 and *R*^2^ = 0.74, respectively). The reason the *E*_B_(N 1s) were more affected by FS effects than *E*_B_(S_anion_ 2p) was investigated further, and it will be discussed below.

The deviations from the *y* = *x* line in these plots are due to final-state effects. However, these deviations are small in comparison with the spread of the values: in Figure 2a (for S 2p core levels) the maximum absolute deviation is 0.19 eV, and the mean absolute deviation is only 0.04 eV, in a range of 1.3 eV. In Figure 2b (for N 1s core levels), the maximum absolute deviation is 0.54 eV, but the mean absolute deviation is only 0.09 eV, in a range of over 5 eV.

The conclusion about the absence of strong FS effects is important because it means that XPS *E*_B_ can be related directly to the ground-state electronic structure of the IL, which is the primary reason we turn to XPS to study these systems. Furthermore, it facilitates our theoretical study of the distribution of core-level binding energies in the IL bulk liquid because calculations in the IS approximation are computationally cheaper and much simpler: one single-point calculation is enough, whereas in the FS approximation, individual calculations explicitly including a core hole at each atom in the liquid are required. In what follows, the reported binding energies were obtained in the IS approximation, unless otherwise stated.

**Figure 3:**
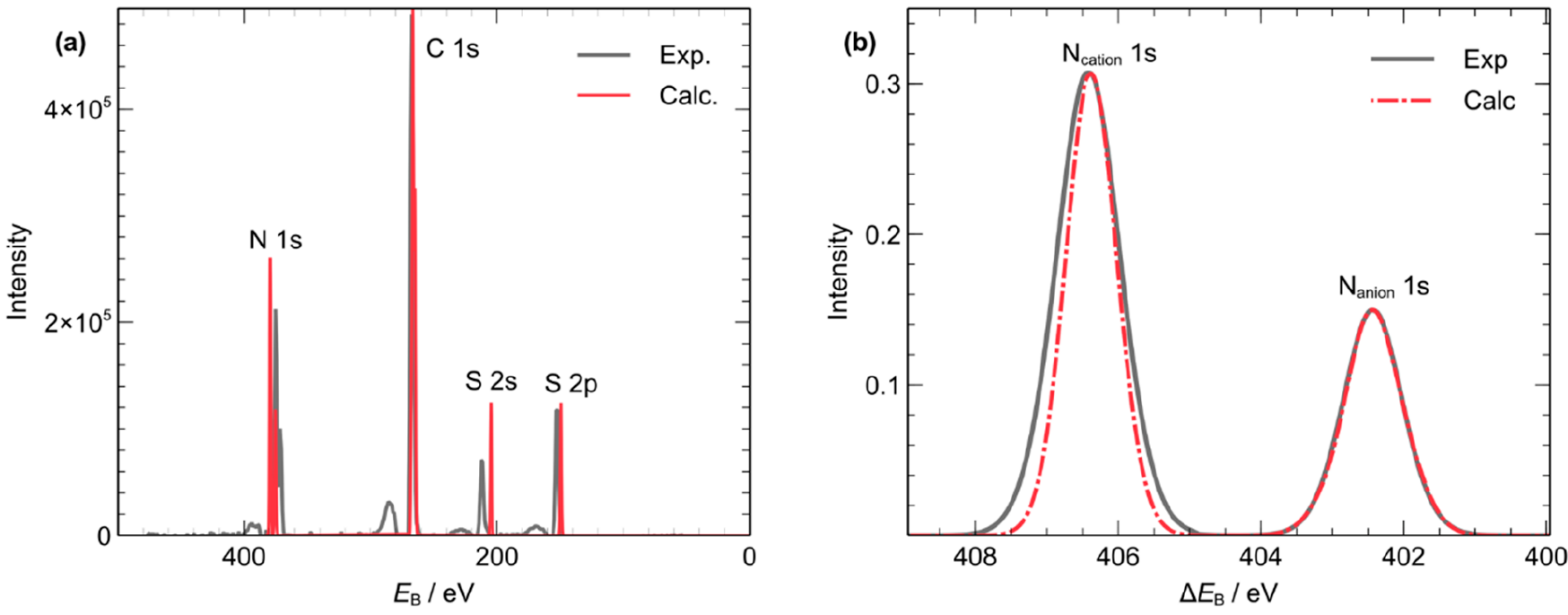
Initial-state effect calculations. (a) Experimental survey scan (gray) overlaid with the calculated survey scan (red). Calculated scan was shifted by +21.2 eV, and intensities were corrected using the C 1s peak. The calculated peaks were broadened with a width of 1 eV to mimic experimental broadening. (b) High-resolution scan of N 1s peaks. The calculated peaks were broadened with a 0.7 eV width to mimic experimental broadening.

Having established the validity of the IS approximation, the accuracy of the calculated distribution of *E*_B_(core) was tested against experiment. The experimental survey XP spectrum was plotted and compared against our calculated survey spectrum (Figure 3). Quantitative and qualitative analyses of neighboring peaks showed the overall accuracy of the computed results to be high, despite the omission of FS effects. Most *E*_B_ separations are within a 3.5% deviation from experiment. In particular, a high-resolution scan comparison shows Δ*E*_B_ = *E*_B_(N_cation_ 1s) – *E*_B_(N_anion_ 1s) is 4.1 eV in experiment and 4.0 eV in calculated peaks (Figure 3b), which are in excellent agreement. The only significant discrepancy in the survey scan plot is a calculated 55.2 eV separation between S 2s and S 2p levels, whereas in experiment the separation was 64.2 eV; this is almost a 9 eV change between the two sets of results, or a 14% error.

**Figure 4:**
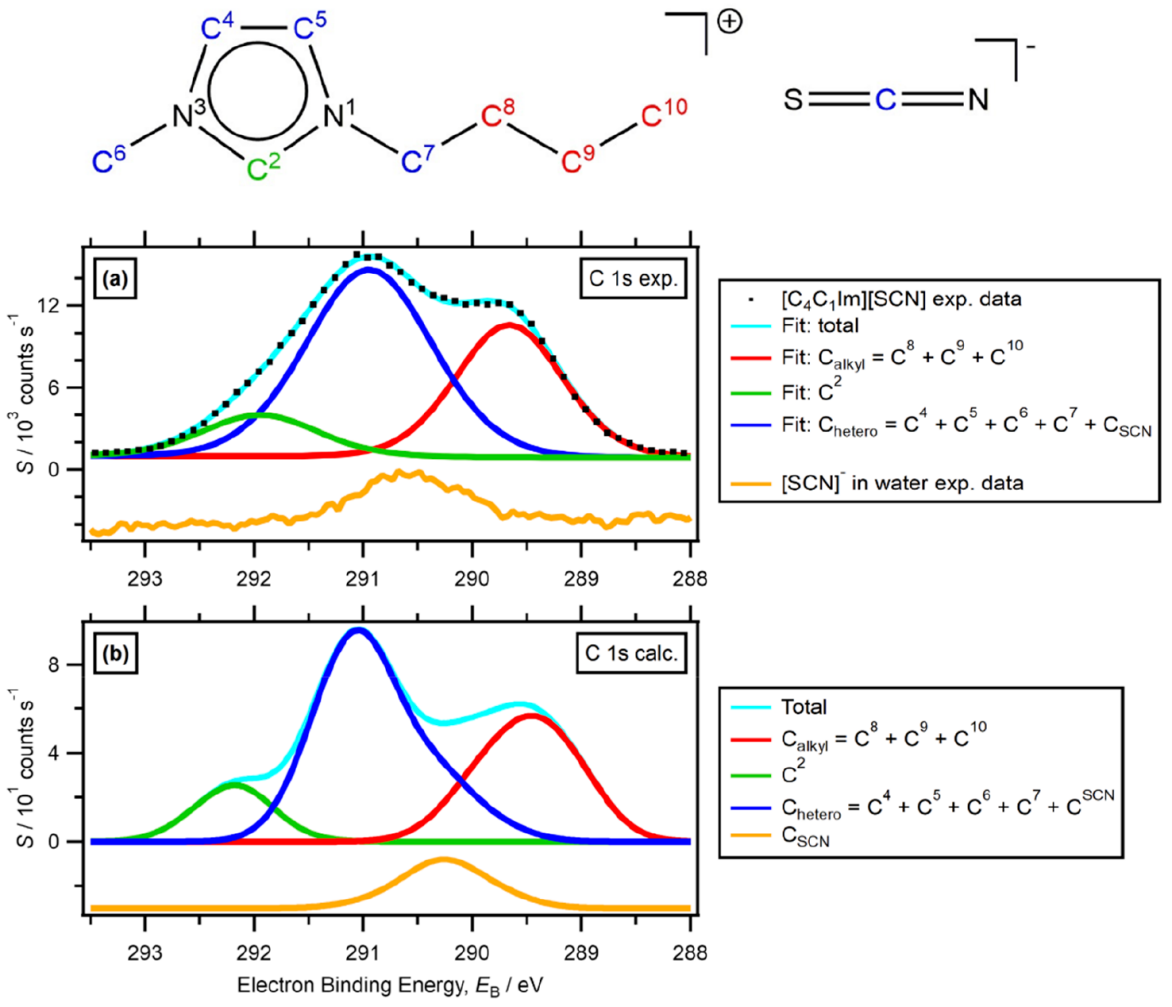
C 1s XPS. (a) Experimental C 1s peak fitting model for [C_4_C_1_Im][SCN] published in ref (93); fitted with peaks for C_alkyl_, C_hetero_, and C^2^. C_hetero_ is likely to contain contributions from the C_anion_ peak, increasing its FWHM. Experimental peak of C_SCN_ from K[SCN] in water. (b) Peaks calculated in this work, with a width function of 1 eV (see Section 2.5 for more details), separated into C_alkyl_, C_hetero_, C^2^, and C_anion_. Calculated C_SCN_ extracted for comparison to experimental *E*_B_. Charge referencing for XP spectra is explained in Section 2.1.

A comparison of C 1s peaks further confirmed the success of the IS calculations in replicating experimental measurements. Our new experimental data have shown the position of the C 1s anion peak from [SCN]^−^, previously unidentified, alongside a fitting model used for the C 1s peak (Figure 4a).^109^ Visual comparison with calculated peaks (Figure 4b) of the same species shows very good agreement—each peak position is matched within 0.1 eV. The experimental FWHM of C bonded to a single N (blue, C_hetero_) is larger than the calculated, due to the unresolved [SCN]^−^ contribution in the experimental fitting.

### 3.2 Peak Broadening

In the DFT calculations, instrumental and lifetime broadening are not simulated. A comparison between measured and calculated data of N_anion_ 1s and N_cation_ 1s peaks shows a very good N_anion_ 1s broadening match and a good N_cation_ 1s match (Figure 3b). A visual assessment of the same plot, but with a 0.5 eV width function, shows that the peaks do not match as well, and some asymmetry is present (ESI Figure S1). Experimental broadening for this data set is estimated as apparatus contributions of ∼0.45 to 0.65 eV and lifetime contributions of 0.054 and 0.115 eV for S 2p and N 1s, respectively, totaling ∼0.5 eV;^110,111^ charging contributions are negligible.^109^ Using an apparatus broadening value of 0.65 eV, the calculated structural broadening values were found to be in the range of 0.55 to 0.82 eV.

Testing increased sample sizes did not increase the N_cation_ 1s peak width to provide a better match to the experimental width, and only a slight improvement in peak symmetry was noted. There are two possible explanations for the slight peak width discrepancy in the spectrum: (i) the AIMD calculation is lacking accuracy in the disorder description of the system, or (ii) the experimental broadening value is underestimated. As the experimental broadening value is not an exact estimate and is likely to fluctuate between calibration and successive experiments, we believe the latter is the most likely source of the slight discrepancy.

### 3.3 Effects of Intraion and Interion Interactions on the Core-Level Binding Energies

**Figure 5:**
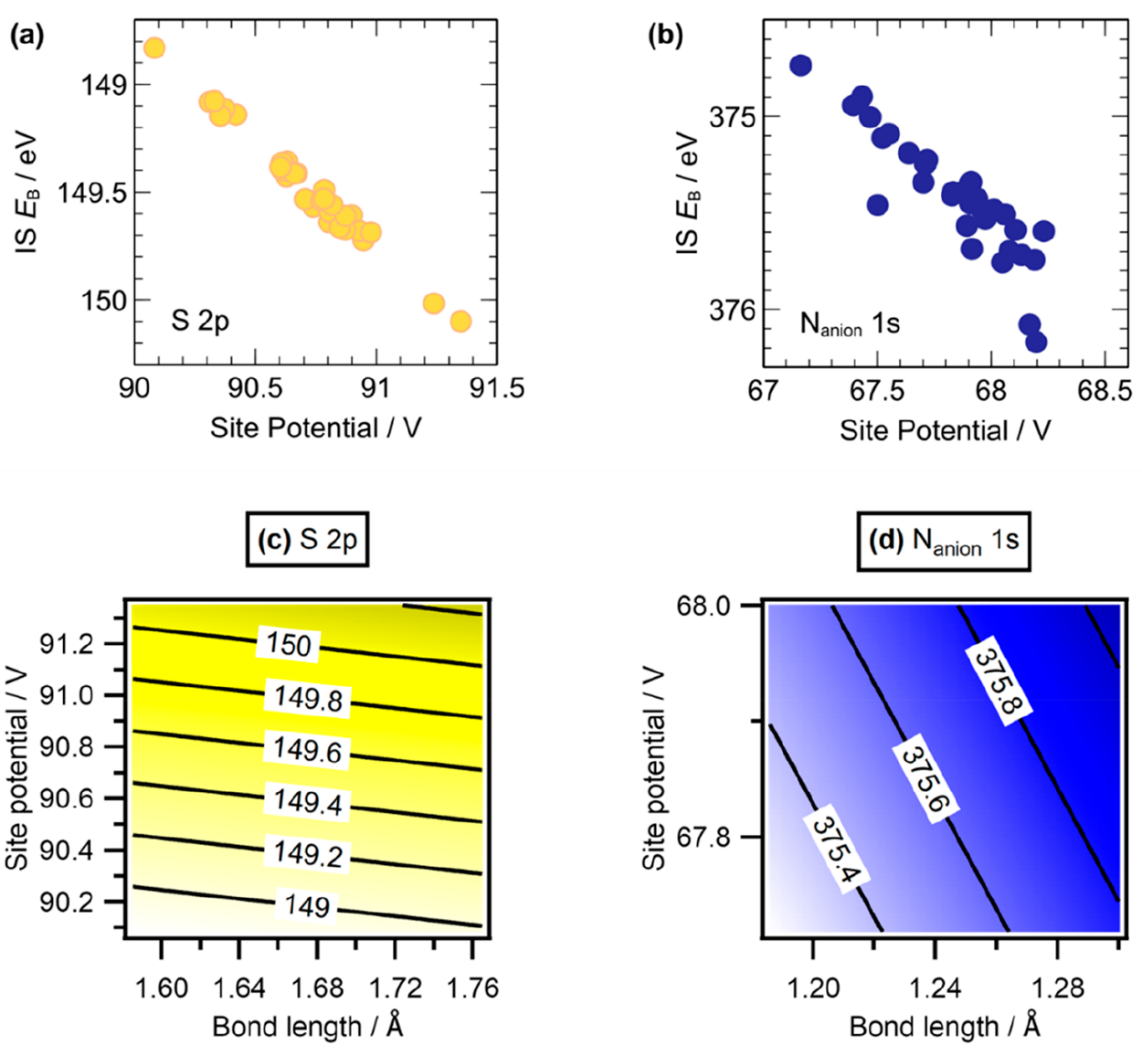
Correlation of IS *E*_B_ with site potential for (a) S 2p (yellow) and (b) N_anion_ 1s (blue). Contour maps of bilinear prediction models for (c) S 2p (yellow) and (d) N_anion_ 1s (blue) with *E*_B_ being a function of both the site potential and the bond length.

Intraion interactions were analyzed through comparison of S–C bond length to the relevant *E*_B_(S_anion_ 2p) and N–C bond length to *E*_B_(N_anion_ 1s). Both plots produced a weak linear correlation (ESI Figure S2). Visual assessment and the RDFs for the analysis of short-range interactions did not produce a clear pattern of short-range interactions in relation to *E*_B_, and this was not investigated further. Neither short-range nor intraion effects were found to be the dominant influence over *E*_B_ fluctuations. Longer-range interactions were characterized by calculating the site potential at each atom. The site potential is defined in VASP as the average of the electrostatic potential in the core region of a given atom. This site potential is affected by both short-range and long-range interactions. Plots of site potentials against *E*_B_ for each atom clearly demonstrated that *E*_B_(S_anion_ 2p) correlates almost perfectly with site potential, with very little deviation (Figure 5a). Although *E*_B_(N_anion_ 1s) also correlates very well with site potential, the deviation is slightly higher than for S 2p (Figure 5b). We successfully interpreted these patterns by further comparison with internal bond lengths in the form of a multiple regression model for *E*_B_, based on both site potential and bond length values. The multiple regression produced a new model for *E*_B_ prediction (Figure 5c,d) which was compared against the actual calculated *E*_B_. The model was found to be highly accurate, with a root-mean-square deviation (RMSD) of only 0.01 and 0.02 eV for S_anion_ 2p and N_anion_ 1s, respectively. In comparison to the linear regressions, which have RMSD values of 0.03 and 0.10 eV, respectively, accuracy is increased substantially, particularly for N_anion_ 1s.

**Figure 6:**
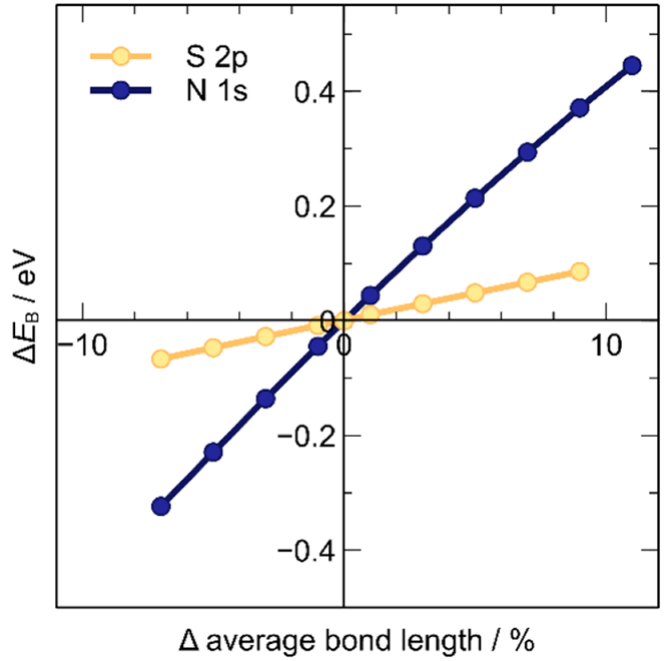
Plot of *E*_B_ change with variation of internal bond length in the anion [SCN]^−^. Bond S–C for S_anion_ 2p and N–C for N_anion_ 1s.

The strong correlation of *E*_B_(S 2p) with site potential was assumed to be the result of the large, highly polarizable nature of the atom.^112^ The electron cloud is understood to be more susceptible to the influence of collective electrons in a disordered bulk IL system than an atom like nitrogen, which is smaller and denser than sulfur. The valence and core electrons of a nitrogen atom were expected to be influenced more strongly by the N–C internal bond length than the sulfur atom is by the S–C internal bond length, due to the higher electron density in the N–C bond. Calculations found that when the S–C or N–C bond was altered in turn, the *E*_B_(N_anion_ 1s) undergoes higher energy changes with the same % bond length variation (Figure 6).

The finding that *E*_B_ correlates very well with site potential shows that single ion pair studies are insufficient to describe the structure and interactions within a real IL system. Single ion or ion pair calculations are, by definition, based on the assumption that short-range intramolecular interactions dominate *E*_B_ broadening. Gas-phase models seem to overestimate the cation–anion interaction when the long-range structure is missing.^113^ A slight improvement to this is an implicit solvent model or ionic pair “clusters”; however, these models cannot explicitly simulate long-range disorder of the liquid system. Focusing on a single ion pair increases chances of error when calculating *E*_B_. This study found an ∼1 eV range in *E*_B_ fluctuations for a single atom type (Figure 2). A single ion pair, especially when optimized, could fall within the extremes of this range, rather than in the middle, or average of this range of *E*_B_. Particularly when resolving shifts of <0.5 eV, this value has been shown to be within our error margin, and any findings related to a shift of this size may be entirely insignificant. This would likely result in inaccurate conclusions on the bulk system.

## 4 Conclusions

This study found a strong correlation between experiment and DFT calculations, demonstrating for the first time that IS contributions were the primary contributor to *E*_B_ values in the IL [C_4_C_1_Im][SCN]. This system is representative of a wide array of ILs, suggesting that IS effects would be the primary contributor to most ILs. Our finding suggests that either FS effects are negligible or are similar across all atoms, so do not contribute to peak separations. Furthermore, we confirm that the effect of the anion on the cation, observed using XPS for many ILs, was driven by an IS effect. We confirmed that *E*_B_ was closely linked to site potential in the anion of [C_4_C_1_Im][SCN]. The site potential is influenced by all (both short- and long-range) interactions of the bulk system. We found no evidence to show that short-range, intermolecular interactions are sufficient on their own to describe the liquid phase *E*_B_ variation. Site potential did not offer a perfect correlation with *E*_B_. The discrepancy was found to mostly correspond to intramolecular bond length fluctuations. With both site potentials and internal bond lengths, we produced a predictive model for *E*_B_, which was found to be highly accurate for this IL.

## Supporting Information Available

Plot of high-resolution N 1s scan with 0.5 eV broadening; plots of IS *E*_B_ vs internal bond length for S 2p and N 1s (PDF)
