# Supplementary material for: Understanding X-ray Photoelectron Spectra of Ionic Liquids: Experiments and Simulations of 1-Butyl-3-methylimidazolium Thiocyanate
Source: J Phys Chem B. 2022 Dec 1;126(49):10500–9. doi: 10.1021/acs.jpcb.2c06372 (PMC9761679; doi:10.1021/acs.jpcb.2c06372)
Supplement: Supplementary file 1 — jp2c06372_si_001.pdf [file jp2c06372_si_001.pdf]

Understanding X-ray Photoelectron Spectra of Ionic Liquids: Experiments and Simulations of 1-Butyl-3-Methylimidazolium Thiocyanate

Ekaterina Gousseva,[a] Scott D. Midgley,[a] Jake M. Seymour,[a] Robert Seidel,[b] Ricardo Grau-Crespo,[a] and Kevin R. J. Lovelock[a*]

[a] *Department of Chemistry, University of Reading, Reading, RG6 6DX, UK.*

[b] *Helmholtz-Zentrum Berlin für Materialien und Energie (HZB), Berlin, Germany.*

*Contact e-mail: k.r.j.lovelock@reading.ac.uk

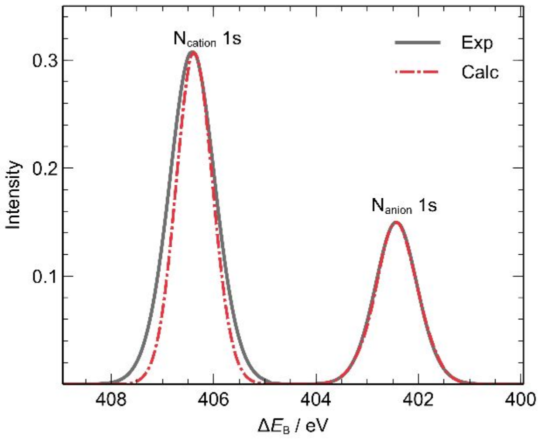

Figure S1 High resolution scan of N 1s peaks. The calculated peaks were broadened with a 0.5 eV width. Computed peaks shifted +27.00 eV for charge referencing to $E_B(C_{alkyl}$ 1s) = 289.58 eV.

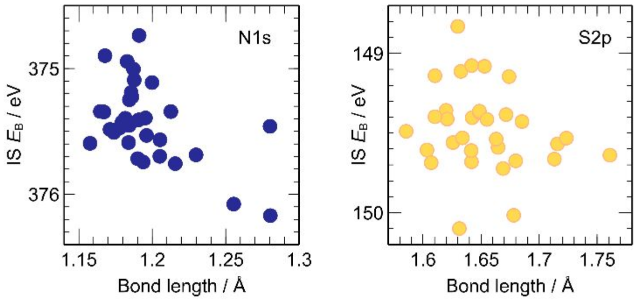

Figure S2 Plot of IS E_B with C-N bond length for N_{anion} 1s (left, blue) and IS E_B with S-C bond length S 2p (right, yellow).
